# Congregational Size and Attitudes towards Racial Inequality among Church Attendees in America

**DOI:** 10.3390/rel6030781

**Published:** 2015-06-25

**Authors:** Ryon J. Cobb, Kevin D. Dougherty, Jerry Z. Park, Samuel L. Perry

**Affiliations:** 1 Davis School of Gerontology, University of Southern California, 3715 McClintock Ave., Los Angeles, CA 90089, USA; 2 Department of Sociology, Baylor University, One Bear Place #97326, Waco, TX 76798, USA; 3 Department of Sociology, The University of Chicago, 5801 S Ellis Ave, Chicago, IL 60637, USA

**Keywords:** Racial attitudes, congregation size, church attendance, racial inequality

## Abstract

Research suggests that congregational characteristics are associated with the racial attitudes of American churchgoers. This study examines the relationship between congregational size and beliefs about the Black/White socioeconomic gap among religious adherents.

**Method:**

Drawing upon data from the General Social Survey and the National Congregations Study, we fit binary logistic regression models to estimate the association between congregational size and Americans’ explanations of Black/White economic inequality.

**Results:**

Findings reveal that attendees of larger congregations are less likely than attendees of smaller congregations to explain racial inequality as the result of the racial discrimination. The likelihood of explaining racial inequality in terms of personal motivation does not vary by congregation size.

**Conclusion:**

Despite the growing diversity in larger congregations in America, such congregations may steer attendees’ views about racial inequality away from systemic/structural factors, which may attenuate the ability of such congregations to bridge racial divisions.

## 1. Introduction

Despite decades of policy initiatives, race continues to account for disparities in income, unemployment, wages, and wealth in the United States [1–3]. The persistence of racial inequalities over the past four centuries presents a considerable dilemma for both scholars and non-scholars [4]. Specifically, how do Americans explain persistent economic gaps between Blacks and Whites in the United States? Uncovering the factors that affect Americans’ understanding of socioeconomic disparities between Blacks and Whites in the United States is important because prior studies have demonstrated strong links between racial attitudes and support for racial race-based policies as well as government spending on social policies among states [5–7].

Over the past five decades, a number of studies have sought to uncover the factors that shape Americans’ beliefs about race-related issues [8]. Given that many Americans view racial inequality as a moral issue, a growing body of literature in this area focuses on the role of religion in shaping Americans’ attitudes toward racial inequalities [9]. Prior research has uncovered statistically significant associations between religion (e.g., religious affiliation, religious service attendance, fundamentalist religious beliefs) and racial attitudes [10–12]. Nevertheless, researchers have yet to investigate the possible association between congregational size and racial attitudes. This neglect is curious, given ongoing attention to the roles of religious institutions in shaping racial attitudes. This neglect also occurs despite evidence that an increasing percentage of Americans are attending religious services in larger congregations [13,14].

The present study adds to literature on religion and racial attitudes by investigating links between congregational size and beliefs about the Black/White socioeconomic gap among church attendees in the United States. Though we are not the first to examine links between religion and outcomes related to racial inequality, there are key differences between the current study and prior works on this issue. First, to date, our understanding of religious differences and racial attitudes among Americans has come mainly from case studies and/or *individual-level* survey data. As a result, we lack systematic information about whether and/or how structural features of religious *congregations* may shape racial attitudes. In order to overcome the limitations of individual level data, we took advantage of a unique dataset—the National Congregations Study (NCS) linked with individual responses from the General Social Survey (GSS)—to investigate the association of religion and racial attitudes. Second, rather than focus on associations between religion and racial attitudes within one religious group, our study examines if the relationship between congregational size and racial attitudes is independent of other known individual and congregational correlates. This study proceeds as follows. We provide a brief review of the current literature relevant to this study and lay out our hypotheses. The remainder of our paper shows and discusses the estimated association between congregational size and beliefs about racial inequality among religious adherents in the United States using an analysis of the GSS and the NCS.

## 2. Background

The relationship between religion and racial attitudes continues to garner attention from researchers in the sciences and humanities as well as from the popular media. Most studies in this area have focused on the relationship between religious denominations and beliefs about racial inequality [15,16]. For instance, in a well-cited work, Emerson and colleagues [17] showed that denominational affiliation accounts for variations in racial attitudes. In comparison to non-evangelical Whites, evangelical Whites were more likely to deny that racial discrimination and a lack of educational opportunities undergird Black/White disparities in socioeconomics. Emerson and colleagues also found that White evangelicals were more likely than non-evangelical Whites to attribute Black/White socioeconomic disparities to blacks’ lack of perceived motivation. Other studies in this area also highlight the role of other *institutional characteristics* in accounting for variations in racial attitudes among religious adherents in the United States. For example, prior studies have found that the size of an organization’s racial minority population accounts for variations in racial attitudes, while other studies have shown the importance of other institutional characteristics such as the ability of congregations to serve as “affinity havens” [18–20].

## 3. Congregational Size as a Predictor of Anti-Structural and Individualist Understandings of Racial Inequality

Though we know of no work that directly explores the association between congregational size and racial attitudes, there are several reasons to expect that congregational size is a statistically significant predictor of racial attitudes in the United States. Sociologists have long suggested that larger organizations require increased differentiation and bureaucratization. For instance, Simmel [21] argued that smaller organizations were more effective at maintaining a high degree of social solidarity, shared beliefs, and egalitarian dialogue among individuals. Other studies have demonstrated that (a) affiliation with larger organizations requires the reinforcement of certain practices to maintain administrative and communicative efficiency and (b) increasing organizational size creates differentiation at decelerating rates, which in turn, leads to bureaucracy, separation, and un-involvement [22,23]. Consistent with the claim that larger organizations are associated with decreases in social solidarity, beliefs, and egalitarianism, some sociologists of religion [24] have argued that adherents in larger congregations are more prone to “free-riding”. Free-riding is a condition in which individuals may benefit from a collective goods produced by organizations they did little to help produce [25]. A free-rider in congregation is the individual who attends the potluck dinner without bring a meal. In large congregations, aggregate levels of participation are reduced by the presence of free-riders [26,27]. Taken together, these studies suggest that larger congregations are less effective at maintaining social solidarity and shared beliefs. For a congregation to change the racial attitudes of an individual, it must be a salient environment for an individual. Tight social bonds are a defining feature of strong congregations [24,26]. The absence of these bonds renders a congregation less likely to change prevailing views and values. Large congregations, especially very large congregations, may suffer this fate. They may attract thousands to weekly services without ever helping most of those gathered become more than a crowd of strangers. Since social isolation and anti-egalitarian attitudes have been shown to increase support for anti-structural and individualist explanations of racial inequality [28], we hypothesize the following:

Hypothesis One: Individuals who attend religious services in larger congregations will exhibit support for anti-structural and individualist understandings of racial inequality.

## 4. Congregational Size as a Predictor of Structural and Anti-Individualist Understandings of Racial Inequality

An *alternative* perspective is that attending religious services in larger congregations is associated with stronger levels of support for structural rather than individualist attitudes toward racial inequality [29–32]. Specifically, the “size as an asset” view of the relationship between congregational size and individual level beliefs/attitudes contends that larger congregations offer higher quality resources that meet the specialized needs of congregations. Several supporting reasons appear. First, Stonebraker [33] found that “a denomination comprised of large congregations will produce more total ministry than a similar-sized denomination comprised of smaller congregations”. Second, these specialized ministries or small groups make the religious experiences within a larger organizational more manageable, as small groups provide individuals with social relationships. Given this, it should come to no surprise that researchers have found that small groups are associated with increasing a sense of congregational embeddedness s, more frequent attendance, and higher rates of involvement among religious affiliates [34,35]. Third, the presence of small groups, and specifically, the social relationships within organized religious life have been important in breaking down racial barriers within very large congregations [36]. For instance, Edwards [37] discovered that membership within a congregational small group was one important factor that led individuals to form the type of social relationships needed to shape racial attitudes. Finally, researchers have drawn links between congregational size and racial diversity. This interest has largely come in the wake of Scott Thumma’s influential works on megachurches, or Protestant congregations with at least 2000 weekly attendees [38]. In contrast to the popular belief that very large Protestant congregations are overwhelmingly comprised of individuals from similar racial or ethnic backgrounds, Thumma found that fifty-five percent of megachurches made intentional efforts to become diverse in 2005. At the same time, a little more than thirty percent of all megachurches identified as a multiracial congregation, meaning that no one racial group comprises more than 80 percent of membership. This is important because, as Yancey [39] and others have reasoned, multiracial religious congregations seem to exhibit the ideal contact conditions of equal status, common goals, cooperative tasks, support from authorities, and opportunities to develop intimate relationships that result in positive racial attitudes. The voluntary nature of congregations tends to attract those with similar ideologies and statuses. Members have incentive to work together for religious or congregational goals through involvement in small groups, Bible classes, workshops and other ministry activities, all of which provide opportunities for friendship formation. Friendships across racial groups can help challenge individualist ideas about racial/ethnic differences and highlight structural factors that perpetuate inequality in society. In this sense, larger congregations may be associated with stronger support for structural and anti-individualist racial attitudes.

Hypothesis Two: Individuals who attend religious services in larger congregations will exhibit support for structural and anti-individualist understandings of racial inequality.

## 5. Data and Methods

Data for these analyses are drawn from two sources: the GSS and the NCS. We use individual-level data from the 1998 and 2006 GSS, nationally representative cross-sectional survey of U.S. adults conducted by the National Opinion Research Center. Since 1994 the GSS has sampled roughly 3000 persons in even-numbered years. We use only data from the 1998 and 2006 GSS because in these years the survey included two extensive special modules of items on religion and spirituality. These individual-level GSS data are combined with data from the 1998 and 2006 NCS, a survey of a nationally representative sample of religious congregations in the United States. The 1998 and 2006 GSS included a set of items asking respondents who say they attend religious services at least once or twice a year to report the name and location of their religious congregation [40]. This procedure generated a nationally representative sample of American congregations. Because each NCS congregation was nominated by at least one GSS respondent who attended that congregation, congregation-level data gathered by the NCS can be linked to individual-level data collected from the GSS respondent who attends that congregation. This linkage enables us to examine the effects of congregational characteristics on individuals. The response rates for the NCS were 80 percent in 1998 and 85 percent in 2006. The response rates for the GSS were 76 percent in 1998 and 71 percent in 2006. The GSS/NCS data are ideal for the goal of our study, which is to test claims regarding general associations between congregational size (as reported by clergy or other church personnel) and racial attitudes (as reported by members). Since the GSS/NCS does not draw on samples of multiple individuals within congregations, it is not surprising that 85 percent of our respondents are the only person in the sample from their congregation. Multi-level modeling with data that contain small sample sizes at different levels of analysis tends to bias results. Therefore, we did not use multilevel modeling in this study.

### 5.1. Dependent Variables

Our dependent variables are based on the following questions within the GSS: On the average, blacks have worse jobs, income, and housing than white people. Do you think these differences are:

- Mainly due to *discrimination.*
- Because most blacks have less *in-born ability* to learn.
- Because most blacks don’t have the *chance for education* that it takes to rise out of poverty.
- Because most blacks just don’t have the *motivation* or will power to pull themselves out of poverty.

Respondents answered Yes or No to each statement (A through D). Following previous research, structurally-oriented racial attitudes attribute economic gaps between Blacks and Whites to systemic deficiencies within social structures and institutions. Therefore, respondents who adhere to structural explanations are those who answered affirmatively to question A (mainly due to discrimination) and/or C (lack of educational opportunities). We created separate dummy variables for discrimination (coded 1 if yes and 0 if no) and education (coded 1 if yes and 0 if no). Individualist explanations for racial inequality contend that blacks’ personal choices (e.g., government dependency) and/or innate inferiority contribute to persistent racial economic inequality. For our individualist measure, respondents who selected lack of motivation (option D) as an explanation for the Black/White socioeconomic gap were coded as 1, with those not selecting this explanation coded as 0. We exclude option B from our analysis, since less than six percent of respondents in our survey responded yes to this question.

### 5.2. Independent Variables

The primary independent variable in this study is congregational size. The NCS asked, “How many persons—counting both adults and children—would you say regularly participate in the religious life of your congregation—whether or not they are officially members of your congregation?” Because congregation size is highly skewed, we used the logged number of regular participants in our statistical models.

### 5.3. Control Variables

Prior studies have shown that a number of social factors are associated with racial attitudes [28]. Therefore, included a variety of control variables to account for these factors. Our study is necessarily restricted to individuals who identify with a religious congregation. We also control for Catholic churches and congregations from other faiths outside Protestant and Catholic Christianity. Protestant churches serve as the reference group in statistical analyses. At the individual level, we control for race/ethnicity, age, education, gender, political ideology, home ownership, church attendance, and region. The GSS asks respondents to identify their racial classification based on three options: Black, White, and Other Race. Following prior works [41], we recoded this variable to divide the racial categories into four dummy codes: Black, White, Hispanic, and Other Race. In this case, respondents in the “other race” category are individuals who selected the GSS’s “other race” option and do not claim any Hispanic ancestry.

Table 1 presents descriptive statistics for predictor variables in our analysis. Our measure for age captures all respondents from age 18 to 89. Education is a continuous variable that captures years of education. Female is a dummy variable coded 1 if female and 0 if male. Political ideology is measured on a 7-point scale, ranging from 1 = extremely liberal to 7 = extremely conservative. We used an ordinal measure for church attendance that ranges from 1 = those who attended religious services once or twice in the past year to 5 = those who attended services more than once a week. We measure region using dummy variables for Midwest, Northeast, West, and South. Since prior studies have shown that residing in the South is associated with racial attitudes, South is the reference category in our statistical models. Given that we are using two years of GSS data, we dummy coded the variable for year such that 1998 is coded as 1 and 2006 is coded as 0.

### 5.4. Statistical Procedure

To test the effect of congregation size, we estimated a series of logistic regression models for the three dependent variables. Tables 2 and 3 report results for structural explanations of racial inequality. Table 4 repeats the analysis for individualist explanations. We report the logistic regression results as odds ratios in all tables.

## 6. Results

Table 2 presents the odds ratios predicting discrimination as a source of the Black/White socioeconomic gap. The logged number of regular attendees in a congregation is inversely related to the likelihood that respondents affirm discrimination as an explanation for racial inequality. A one unit increase in the logged number of regular attendees is associated with a 15 percent decrease in the odds of supporting racial discrimination as an explanation for racial inequality. Race/ethnicity, age, gender, political ideology, and region are statistically significant predictors of racial discrimination as an explanation for Black inequality. Blacks and Hispanics are more likely than Whites to hold this view, as are older adults, women, and respondents living in regions outside of the South. In contrast, holding conservative political views is associated with decreased support for this view.

Table 3 presents odds ratios predicting whether respondents affirm the education explanation for the Black/White socioeconomic gap. In this model, the logged number of regular attendees is not significantly related to support for the education option. That is to say, congregation size seems to have no bearing on the belief that a lack of educational opportunities is the root of racial inequality. Other significant correlates to this explanation are race/ethnicity, education, political ideology, and region. Blacks, more educated respondents, more politically liberal respondents, and non-southerners are more likely to attribute black/white socioeconomic differences to blacks’ lack of educational opportunities.

Table 4 presents odds ratios predicting whether respondents affirm motivation as an explanation for the Black/White socioeconomic gap. The logged number of regular attendees is a statistically insignificant predictor of support for this view. Blacks are less likely than Whites to support this view, whereas Hispanics and other races are actually more likely than Whites to do so. Older and more educated respondents, as well as interviewees that are more politically conservative respondents also affirm motivation as a reason for racial inequality. Women, more education, and residents outside the South are associated with a rejection of motivation to account for Black/White socioeconomic differences.

## 7. Discussion

Our primary aim in this study was to investigate the association between congregational size and attributions of Black impoverishment among religiously active Americans. We proposed competing hypotheses. Our results reveal that as congregation size increases, the odds of attributing Black/White socioeconomic differences to racial discrimination decrease. That is to say, individuals who frequent larger congregations exhibit *less* support for the structural view that racial discrimination is a source for economic disparities between Blacks and Whites in the United States. There was, however, no association between congregational size and the other measure for structuralist racial attitudes (lack of educational opportunities) used in this study. If persons in larger congregations shy away from some structural explanations for inequality, it follows that they would align themselves with more individualist explanations. This expectation was *not* supported by our findings. The logged number of regular attendees had no association with the opinion that lack of motivation accounts for Black/White socioeconomic differences. Hence, we find partial support for our first hypothesis. Persons in larger congregations hold racial attitudes that are somewhat anti-structural but not individualist, as seen in our results, structural and individualist thinking about race do not exist as opposing poles on an ideological continuum. They are independent orientations. Nevertheless, the pronounced conundrum of largeness is noteworthy because more churchgoing Americans than ever before are attending larger congregations. Moreover, if this trend continues, an increasing percentage of religious Americans may report more anti-structural understandings of racial inequality.

## 8. Conclusions

Our findings lead us to several suggestions for future research. One recommendation is to explore the possible mediators and moderators of links between congregational size and racial attitudes. For instance, prior studies suggest that larger congregations may face greater difficulty than previous observers have recognized in fostering perceptions of social warmth and intimacy. Members of big congregations report significantly lower levels of anticipated support than members of other congregations, and this disparity is not offset in congregations with more vibrant and varied small groups, the availability of special-purpose groups to join, or opportunities for informal sociability before and after worship services [27]. Yet, prior studies also indicate that small groups offset some of that effect, by providing individuals in larger congregations with an opportunity to foster social relationships, which in turn, increases feelings of social embeddedness [20,42]. Since social embeddedness and has been shown to shape racial attitudes, some of the association between congregational size and racial attitudes may be explained by the presence of small groups within a congregation. Additional research is necessary to test if and how congregational small groups moderate racial understandings. In addition, future studies to examine the race/ethnicity of respondents and the racial/ethnic composition of congregations as moderating influences on the relationships between congregation size and racial attitudes. Larger congregations are often more racially diverse than smaller congregations [43]. Marti’s [20] ethnographic research on multiracial congregations suggests that, within these diverse religious contexts, ethnic identities can be transcended (or at least minimized) in order to unite congregants around a shared religious identity. In addition to Marti, other scholars [9] have identified that positive interracial relationships within multiracial congregations helped to alleviate racial tensions and promote more progressive attitudes toward racial issues, particularly among Whites.

Finally, we propose a continued examination of ethnic/racial interaction within congregations that have more than 2000 average weekly attendees (megachurches). The absence of strong in-group bonds may be at work to preserve anti-structural attitudes toward racial inequality in these settings. So while efforts to diversify in very large congregations show evidence of the kind of institutional support and common goal-making conducive to reducing racial prejudice [44–47], it is possible that the main ingredient, contact among individuals across racial lines, is lacking [48]. Given the growing number of individuals in very large congregations, it is important to consider the potential of these religious settings for altering attitudes toward structural racism. Based on our findings, when it comes to race relations, bigger (congregations) may not be better. In fact, in a supposedly post-racial era, the specter of prejudice persists among larger crowds of the faithful.

## Figures and Tables

**Table 1:** Descriptive statistics on selected variables (*N* = 1712), General Social Survey/National Congregations Study (*N* = 1712).

Variable	Mean	S.D.
Number of Weekly Attendees (logged)	6.08	1.43
Evangelical	0.35	0.48
Mainline Protestant	0.23	0.42
Black Protestant	0.11	0.31
Catholic	0.28	0.45
Other Faith	0.03	0.17
Black	0.14	0.34
Hispanic	0.08	0.28
Other Race	0.03	0.18
White	0.75	0.43
Age	47.11	16.97
Female	0.61	0.49
Political Ideology	4.28	1.44
Home Owner	0.72	0.45
Church Attendance	5.20	2.00
Midwest	0.25	0.43
Northeast	0.17	0.37
South	0.38	0.49
West	0.20	0.40

**Table 2:** Estimated odds ratios for Americans’ support of racial discrimination as an explanation for the black/white socioeconomic gap, General Social Survey/National Congregations Study (*N* = 1712).

Variables	Odds Ratio	Standard Error	*p*-Value
Logged Number of Regular Attendees	0.85	0.04	0.00
Catholic	1.02	0.16	0.90
Other Faith	0.78	0.25	0.44
Black	2.93	0.48	0.00
Hispanic	1.83	0.39	0.01
Other Race	1.57	0.47	0.13
Age	1.01	0.00	0.00
Education	1.01	0.02	0.66
Female	1.33	0.15	0.01
Political Ideology	0.81	0.03	0.00
Home Owner	0.85	0.11	0.19
Church Attendance	0.99	0.03	0.63
Midwest	1.40	0.20	0.02
Northeast	1.41	0.24	0.04
West	1.62	0.25	0.00
Year	0.98	0.01	0.24
Chi-Squared	151.62		0.00
Pseudo R^2^	0.07		

**Table 3:** Estimated odds ratios for Americans’ support of education as an explanation for the black/white socioeconomic gap, General Social Survey/National Congregations Study (*N* = 1712).

Variables	Odds Ratio	Standard Error	*p*-Value
Logged Number of Regular Attendees	0.98	0.04	0.65
Catholic	0.93	0.14	0.60
Other Faith	0.69	0.21	0.22
Black	1.60	0.26	0.00
Hispanic	1.10	0.23	0.67
Other Race	0.84	0.25	0.56
Age	1.00	0.00	0.13
Education	1.10	0.02	0.00
Female	1.19	0.13	0.09
Political Ideology	0.85	0.03	0.00
Home Owner	0.92	0.11	0.46
Church Attendance	1.00	0.03	0.97
Midwest	1.95	0.26	0.00
Northeast	1.86	0.30	0.00
West	1.87	0.28	0.00
Year	1.00	0.01	0.91
Chi-Squared	108.42		0.00
Pseudo R^2^	0.047		

**Table 4:** Estimated odds ratios for Americans’ support of motivation as an explanation for the black/white socioeconomic gap, General Social Survey/National Congregations Study (*N* = 1712).

Variables	Odds Ratio	Standard Error	*p*-Value
Logged Number of Regular Attendees	0.99	0.04	0.83
Catholic	0.94	0.14	0.68
Other Faith	1.03	0.32	0.92
Black	0.60	0.10	0.00
Hispanic	1.47	0.31	0.07
Other Race	2.23	0.67	0.01
Age	1.01	0.00	0.01
Education	0.90	0.02	0.00
Female	0.79	0.08	0.03
Political Ideology	1.15	0.04	0.00
Home Owner	1.08	0.13	0.50
Church Attendance	0.97	0.03	0.34
Midwest	0.51	0.07	0.00
Northeast	0.72	0.12	0.04
West	0.60	0.09	0.00
Year	1.01	0.01	0.27
Chi-Squared	138.36		0.00
Pseudo R^2^	0.06		
